# Disease-modifying therapies and SARS-CoV-2 vaccination in multiple sclerosis: an expert consensus

**DOI:** 10.1007/s00415-021-10545-2

**Published:** 2021-04-12

**Authors:** Diego Centonze, Maria A. Rocca, Claudio Gasperini, Ludwig Kappos, Hans-Peter Hartung, Melinda Magyari, Celia Oreja-Guevara, Maria Trojano, Heinz Wiendl, Massimo Filippi

**Affiliations:** 1 Department of Systems Medicine, Tor Vergata University, Rome, Italy; 2 Unit of Neurology, IRCCS Neuromed, Pozzilli (IS), Italy; 3 MS Center and Neurology Unit, IRCCS San Raffaele Scientific Institute, Via Olgettina, 60, 20132 Milan, Italy; 4 Neurorehabilitation Unit, IRCCS San Raffaele Scientific Institute, Milan, Italy; 5 Neurophysiology Service, IRCCS San Raffaele Scientific Institute, Milan, Italy; 6 Neuroimaging Research Unit, Division of Neuroscience, IRCCS San Raffaele Scientific Institute, Milan, Italy; 7 Vita-Salute San Raffaele University, Milan, Italy; 8 Department of Neurosciences, San Camillo Forlanini Hospital, Rome, Italy; 9 MS Center and Research Center for Clinical Neuroimmunology and Neuroscience Basel (RC2NB), Departments of Medicine, Clinical Research and Biomedicine and Biomedical Engineering, University Hospital and University of Basel, Basel, Switzerland; 10 Department of Neurology, Medical Faculty, Heinrich-Heine University, University Hospital Duesseldorf, Düsseldorf, Germany; 11 Brain and Mind Centre, University of Sydney, Sydney, Australia; 12 Department of Neurology, Medical University of Vienna, Wien, Austria; 13 Danish Multiple Sclerosis Center, Department of Neurology, Copenhagen University Hospital, Rigshospitalet, Copenhagen, Denmark; 14 Department of Neurology, Hospital Clínico San Carlos, IdISSC, Madrid, Spain; 15 Departamento de Medicina, Facultad de Medicina, Universidad Complutense de Madrid (UCM), Madrid, Spain; 16 Neurology and Neurophysiopathology Unit, Department of Basic Medical Sciences, Neuroscience and Sense Organs, University of Bari “Aldo Moro”, Bari, Italy; 17 Department of Neurology, University Hospital Münster, Münster, Germany

**Keywords:** Multiple sclerosis, Disease-modifying treatments, Vaccine, COVID-19 pandemic

## Abstract

Coronavirus disease (COVID-19) appeared in December 2019 in the Chinese city of Wuhan and has quickly become a global pandemic. The disease is caused by the severe acute respiratory syndrome coronavirus type-2 (SARS-CoV-2), an RNA beta coronavirus phylogenetically similar to SARS coronavirus. To date, more than 132 million cases of COVID19 have been recorded in the world, of which over 2.8 million were fatal (https://coronavirus.jhu.edu/map.html). A huge vaccination campaign has started around the world since the end of 2020. The availability of vaccines has raised some concerns among neurologists regarding the safety and efficacy of vaccination in patients with multiple sclerosis (MS) taking immunomodulatory or immunosuppressive therapies.

## COVID-19

The clinical course of COVID-19 is characterized by three different phases. In the first phase of the disease, the patient is asymptomatic or has mild flu-like symptoms characterized by fatigue, high temperature and dry cough. In this phase the active virus replication takes place and lymphopenia may be present. As the disease progresses, a typical viral pneumonia evolves detectable typically on chest computed tomography as pneumonic infiltrates, so-called ground-glass opacities. The most severe phase of the disease is characterized by a hyper-inflammatory state sustained by the high production of proinflammatory cytokines, the so-called cytokine storm. This leads to sepsis and disseminated intravasal coagulation which may manifest with both multiorgan failure and fatal outcome [1].

The main entry route of SARS-CoV-2 involves the interaction between the viral spike protein and the angiotensin-converting enzyme-related carboxypeptidase 2 (ACE2) expressed by the host cells [2]. The exact mechanisms of antigen presentation and consequent activation of the innate and adaptive immune response are not completely clear. The activation of innate immunity seems to achieve the strong cytopathic properties of virus, following the release of damage-associated molecular patterns and pathogen-associated molecular patterns, giving rise to an excessive production of proinflammatory cytokines. Confirming this hypothesis, a marked increase in circulating inflammatory cytokines, such as interleukin (IL)-1β, IL-6, IL-7 and TNF-α, is observed in patients with more severe symptoms. Moreover, the increased production of such molecules, so-called cytokine storm, correlates with a more rapid progression to acute respiratory distress syndrome [3]. To date, the exact cellular source of the cytokines is not clear. Two scenarios are considered possible and not mutually exclusive. The first is that cells of the innate immunity, directly stimulated by the viral infection, produce inflammatory mediators. The alternative hypothesis holds that the hyperactivation of innate immunity is secondary to hyper-activated T cells [4]. The affection of the central nervous system (CNS) is not uncommon in COVID-19, anosmia, stroke and encephalopathy being the most prominent manifestations [5]. Mechanisms are not yet entirely clear, but Neuro-COVID seems to be associated with an exhaustion of T cells and dedifferentiation of monocytes in the cerebrospinal fluid [6].

As with all viral infections, adaptive immunity is also important in countering SARS-CoV-2 infection. CD4 central memory and CD8 effector memory cells represent the cellular subsets most active against viral SARS-CoV-2 proteins [7]. Dysregulation of T-cell immunity is likely to contribute to disease severity, low T-regulatory subsets and expansion of pro-inflammatory GM-CSF+ CD4+ and IL-6+ CD4+ T cells have been detected in patients with more severe disease [4].

Regarding humoral immunity, the appearance of protective antibodies occurs 7–14 days after the onset of disease. However, it should be considered that the presence of a high IgG response is associated with a more severe disease course, suggesting a detrimental pathogenic antibody-dependent response in the same cases [8].

## Disease-modifyng therapies and COVID-19

Almost all MS patients take immunomodulatory or immunosuppressive drugs to lessen disease activity, severity and to prevent or slow disease progression. Therefore, the current COVID-19 pandemic requires us to understand whether these therapies can increase the risk of infection or worsen the clinical course of the disease [9]. Currently, available evidence does not suggest that MS patients per se develop more severe COVID-19 [10, 11]. A recent international study conducted on a database of over 30,000 patients with MS highlighted how the main risk factors were related to pre-existing clinical conditions, such as comorbidity score ≥ 1, body mass index ≥ 30, and Black/African ancestry, comparable to those seen in the general population [12].

Interferon-β therapies (IFNs) represent the first disease-modifying therapy (DMT) approved for the treatment of MS. Their mechanism of action is pleiotropic, involving the shift of the balance toward the anti-inflammatory Th-2 cells and inhibition of T-cell migration as a result of blockade of metalloproteases and adhesion molecules. The activation of the IFN pathway leads to the induction of several genes, so-called IFN-stimulated genes, many of which interfere with the synthesis of viral proteins [13]. Preliminary data seem to show a certain sensitivity of SARS-CoV-2 to treatment with type-I IFNs, as in vitro IFN I causes an attenuation of SARS-CoV-2, but not SARS-CoV, replication [14]. Another study showed that early IFN therapy is associated with favorable clinical responses in COVID-19 patients [15]. Overall, treatment with IFNs does not appear to increase the risk of infection or worsen the disease course [16, 17], while many studies demonstrate that these molecules reduce the risk of SARS-CoV-2 infection in treated MS patients [12].

Glatiramer acetate (GA) is a synthetic polypeptide containing four naturally occurring amino acids (l-glutamic acid, l-lysine, l-alanine and l-tyrosine), like the myelin basic protein. Although its precise mechanism of action is still unknown, GA has been reported to induce a shift from Th1 to Th2 responses, with an increase in T-regulatory and down-regulation of both Th1 and Th17 cells. The extensive data available from clinical practice do not show an enhanced infective risk in patients taking GA [18], and some studies seem to prove a lower incidence of positivity to SARS-CoV-2 in patients treated with this drug [12]. Hence no greater risk of infection is expected nor any impact on the clinical course of COVID-19 disease.

Fingolimod, the first oral drug approved for MS treatment, is a sphingosine-1-phosphate receptor modulator. It blocks only central memory T cells in lymph nodes, without compromising the circulation of effector memory T cells, and for this reason it generally does not affect the ability to respond to infections [19]. It is still unclear whether fingolimod can negatively affect SARS-CoV-2 infection, however, preclinical data show that sphingosine-1-phosphate receptor modulating drugs reduce the inflammatory pulmonary infiltrate during influenza infection and enhance endothelial barrier function [20].

Teriflunomide interferes with de novo pyrimidine synthesis by specific inhibition of the mitochondrial enzyme dihydro-orotate dehydrogenase (DHODH), an enzyme highly expressed in proliferating lymphocytes. By interfering with pyrimidines synthesis, teriflunomide also inhibits viral replication, as confirmed by in vitro data [21]. Current data from case series and retrospective studies show that severe disease course was observed in none of 79 patients treated with teriflunomide and infected with SARS-CoV-2 [17, 22–24]. Moreover, teriflunomide is even assumed to have possibly beneficial effects because of its antiviral mechanism, and a DHODH inhibiting principle is currently under investigation to prevent COVID-19 morbidity and mortality [25].

Although the therapeutic mechanism of action of dimethylfumarate in MS has not been fully elucidated, it is thought to stimulate Nrf2-pathways with consequent anti-oxidative, anti-inflammatory and cytoprotective effects. Experimental data show that enhancement of the NRF2-pathway reduces macrophage-mediated alveolar damage and induces the expression of antioxidant genes during influenza [26]. Moreover, a recent study demonstrated that dimethylfumarate inhibits SARS-CoV-2 replication and the expression of associated inflammatory genes [27]. Therefore, dimethyl fumarate could have a beneficial anti-inflammatory and cytoprotective action in SARS-CoV-2 infection. Small case series suggest the safety of dimethylfumarate in patients with COVID-19 and normal lymphocyte count, although in the case of lymphopenia a more severe infection cannot be excluded [28].

Natalizumab is a monoclonal antibody that binds to alpha-4 integrin and thereby prevents leukocytes from crossing the blood–brain-barrier. Except for progressive multifocal leukoencephalopathy, natalizumab does not significantly increase the risk of infections [29]. Therefore, it is not expected to increase COVID-19 severity [30]. Most neurological complications during COVID-19 seem to be secondary to indirect mechanisms as cytokine storm, hypercoagulability, multiorgan failure, dysmetabolic states, para- and post-infectious autoimmunity [31]. However, the possible direct invasion of CNS by SARS-Cov-2, a rare but noteworthy condition, must be considered. In this case, the reduction of immunosurveillance in CNS caused by natalizumab could in principle cause severe neurological involvement and sequelae.

Recently, the North American Registry of MS patients described a reduced risk of ICU admission and ventilation in patients treated with fumarate and natalizumab compared to untreated patients [11].

Alemtuzumab is a monoclonal antibody that binds the CD52 glycoprotein and induces antibody-dependent and complement-mediated cytotoxicity. There is an increased infection risk following alemtuzumab therapy, which is dependent on the time of administration of the drug [32]. It is well known that the percentage of infectious complications during therapy with alemtuzumab is closely related to the lymphocyte count and in particular to the lymphopenia that the drug itself induces. Their incidence is, in fact, higher during the first month after drug infusion, and they mostly consist of mild upper respiratory and urinary tract infections, and very rarely systemic infections such as gastroenteritis, diffuse pneumonia and sepsis [33–35]. However, the predominant involvement of adaptive immunity accounts for the low rate of life-threatening infections [36]. The mild disease of the few reported cases of COVID-19 in patients treated with alemtuzumab suggests a prevalent beneficial anti-inflammatory effect of the drug [37–39].

Anti CD20 monoclonal antibodies induce cell lysis by antibody-dependent and complement-mediated cytotoxicity. In addition to B-cell specific subsets of CD20+, T cells are also targeted by the drug. B-depletion has an effect not only on humoral immunity but also on T-mediated responses. Indeed B cells are necessary for maintaining naive CD4+ and CD8+ T-cell homeostasis [40]. It has been reported that anti-CD20 monoclonal antibodies polarize innate immunity cells in a less inflammatory way, thus potentially mitigating the inflammatory response to the virus, and essentially preventing the evolution to severe forms of the disease [41]. It is still unclear, however, whether anti CD20 antibodies elevate the risk of SARS-CoV-2 infection, as reported in a recent clinical study [12]. Duration of exposure might play a role, as suggested by the North American Registry, which found an increased risk of hospitalization in patients treated with rituximab, but not in those treated with ocrelizumab [11].

Probably, the increased rate of infection could be explained by the fact that anti-CD20 antibodies induce an inability to produce, especially close to the drug infusion, an adequate humoral response against the pathogen [42]. The possibility of developing iatrogenic hypogammaglobulinemia should also be considered, which, in addition to increasing the risk of infection, tends to positively affect the rate of reinfection, hindering the development of anti SARS-CoV-2 antibodies after infection with COVID-19 in patients treated with ocrelizumab [43]. Notably, the risk factors that can influence severe COVID-19 disease courses in patients treated with ocrelizumab are the same as in the general population. At the same time, fatal cases of SARS-CoV-2 infection in MS patients treated with ocrelizumab seem to occur at a higher percentage than those occurring during treatment with other approved DMTs [44]. This is probably due to a higher prevalence of Sars-CoV-2 infections in MS patients treated with anti-CD20 compared to other drugs. A recent study conducted in patients with COVID-19 and rheumatic diseases treated with rituximab, another anti-CD-20 drug, demonstrated an increase in the rate of hospitalization in intensive care units and an increase in mortality rate compared to patients treated with other antirheumatic drugs [45]. This evidence seems to be confirmed after correction for possible confounding factors, although, by admission of the authors themselves, patients treated with rituximab generally have a poor response to traditional first-line therapies, together with extra-articular manifestations of disease, chronic use of corticosteroids, and cardiovascular and metabolic risk factors similar to the general population affected by severe forms of COVID-19 disease.

Cladribine is a purine analog that interferes with DNA synthesis. It induces a prolonged lymphocyte depletion more evident for B lymphocytes [46]. Phase-III studies have shown a low infection risk in cladribine-treated patients which may be explained by the fact that patients in treatment with this drug rarely develop severe lymphopenia with lymphocyte ratio less than 500 cells/ml [47]. Since the drug has been introduced only a couple of years ago, few data are available to date. However, given the low impact on innate immunity of cladribine, a higher risk of developing severe COVID-19 is not expected.

## COVID-19 vaccines

Although some drugs cause reduced disease duration and mortality, currently there is no specific therapy effective in all cases of COVID-19 [48]. Therefore, vaccination is the most effective way to counteract the pandemic. The complete genomic sequencing of SARS-CoV-2, available from January 2020, allowed the rapid development of several new-generation vaccines. SARS-CoV-2 is a single-stranded positive-sense RNA virus composed of four main structural proteins: spike protein (S), envelope protein (E), membrane protein (M) and nucleocapsid protein (N). The S protein, located at the outer surface of the virus particles, binds to ACE2 on the cell surface allowing receptor-mediated endocytosis of the virus [49]. As protein S is crucial for the virus to enter the cell, many vaccines use this protein as antigen.

To date (March 2021), 3 vaccines have been approved by EMA in the EU. Two are mRNA-vaccines encoding protein S. They do not contain live materials so they are easier to synthesize and do not have the risk of disease transmission. As mRNA molecules have low transfection efficacy, lipid nanoparticles are used to incorporate them to augment transfection capacity [50]. Once phagocytosed by a cell, lipid nanoparticles are exposed to a low-pH environment in the endosome and the RNA-condensing lipid can pierce the endosome and allow the mRNA molecule to be released in the cytosol.

These vaccines are administered intramuscularly in two administrations 21 days (Pfizer-BioNTech), or 28 days (Moderna) apart.

The third vaccine approved (produced by AstraZeneca) and widely administered in the UK and in many countries of the world is an adenoviral vector-based vaccine. In this vaccine, the double-stranded DNA encoding the coronavirus spike protein antigen is cloned into a viral vector that lacks the ability to reproduce. The viral vector, mimicking a viral infection disease state, produces strong cellular immune responses [51]. Two intramuscular injections are administered 4–12 weeks apart. Other new generation vaccines under study are recombinant protein vaccine, bacterial vector-based vaccine, plasmid DNA vaccine and trained immunity-based vaccine. Traditional inactivated vaccines are also among the candidate vaccines against SARS-CoV-2 (for regular updates please look at London school of hygiene & Tropical medicine—Vaccine tracker https://vac-lshtm.shinyapps.io/ncov_vaccine_landscape/#).

## Vaccines and DMTs

The development of protective immunity over time mainly requires the correct functionality of adaptive immunity played by B and T lymphocytes. Stimulation of B and T lymphocytes by specific antigens leads to their clonal expansion with the formation of memory clones. Memory cells after a re-exposure to the same antigen proliferate rapidly transforming themselves into effector cells. B cells upon activation differentiate into plasma cells which first produce IgM class antibodies and later IgG antibodies.

Safety and efficacy of vaccines against various pathogens in patients with MS are now well established. Several studies have shown that there is no difference in vaccine responses between MS patients and healthy subjects [52]. Conversely, the evaluation of safety and effectiveness of vaccines in MS patients taking DMT is more complex.

Several studies demonstrate that IFNs do not compromise the efficacy of vaccines. No significant differences between patients treated with IFNs and healthy subjects were found in the degree and duration of humoral immunity. Cellular immune response, as measured by the frequency of IFN-gamma secreting T cells in response to influenza antigen, was also similar [53]. Even duration of protective antibody titer against influenza was comparable between patients treated with IFNs and healthy subjects [54]. To date, overall available data demonstrate adequate immune responses to sundry vaccines in MS patients treated with IFNs.

An observational study comparing the immune response to inactivated influenza vaccine between patients treated with GA and healthy subjects showed similar seroprotection in the two groups. The authors detected a lower response in the treatment group, however, the difference was not statistically significant [55]. An efficient immune response was confirmed by a recent prospective study in which patients on GA demonstrated post-influenza vaccination seroprotection rates close to those of the comparator beta-interferon treated group [56].

Teriflunomide showed a modestly diminished rate of immune response to vaccines, however, it does not appear to compromise the achievement of seroprotective antibody levels. Patients treated with teriflunomide generated effective immune responses to seasonal influenza vaccination [57]. Antibody responses to rabies vaccine neoantigen and delayed hypersensitivity to candida albicans, trichophyton and tuberculin were shown to be safe and effective in subjects treated with teriflunomide, although the ratios of post-vaccination to pre-vaccination geometric mean titers was lower in the treated versus the placebo group [58].

An observational study evaluating vaccine responses in patients taking dimethyl fumarate and IFNs revealed comparable vaccine response in both groups [59]. Based on these data and considering the mechanism of action of the drug, a negative effect on vaccinations is not expected.

A blinded, randomized, multicentre, placebo-controlled study of responses to seasonal influenza vaccine and tetanus toxoid booster was conducted in patients receiving fingolimod. Although many patients have shown to develop a valid immune response, the proportion that achieved seroprotective titers was lower in the treatment group than healthy subjects [60]. The reduced response to influenza vaccine during treatment with fingolimod was also confirmed by a further prospective study [55]. Similar results were also observed for the S1P receptor modulator siponimod [61].

A similar immune response to diphtheria-tetanus toxoid and neoantigen Keyhole limpet hemocyanin (KLH) was detected in patients treated with natalizumab and healthy subjects [62]. Conversely, a real-life study and a prospective study evaluating, respectively, H1N1 [54] and seasonal influenza vaccination [55] have provided evidence that an inadequate response to the immunization may occur in some patients treated with natalizumab.

The only small study evaluating the effect of alemtuzumab on 3 vaccines (diphtheria, tetanus, and poliomyelitis vaccine, haemophilus influenzae type B and meningococcal group C conjugate vaccine, and pneumococcal polysaccharide vaccine) demonstrated normal responses, though somewhat blunted for vaccinations within 6 months from last infusion [63]. However, in light of the limited data available, it is recommended to complete vaccinations at least 6 weeks before starting alemtuzumab. The timing of vaccination is further controversial if therapy has been already started. In this case, it is reasonable to wait 3–6 months after the last dose. In fact, after drug-induced depletion, B cells usually return at basal levels at 3–6 months, although 12 months are generally requested to switch to a mature phenotype. Moreover, it should be considered that cellular T depletion tends to persist much longer (about 30 months for CD8+ cells and 60 months for CD4+ cells) [64].

The VELOCE trial investigated the effect of ocrelizumab on different vaccinations. Humoral response to inactivated vaccines studied was detected, although attenuated [65]. Therefore, as some patients may not develop an effective immunization during B-cell depletion it may be useful to delay the first course of ocrelizumab and receive the vaccine at least 6 weeks before infusion. If the risk/benefit ratio supports the need for an urgent (re)treatment, an individualized strategy should be assessed for each patient. For example, if a normal ocrelizumab treatment regimen is scheduled (dosing interval of 6 months) it could be reasonable to administer the vaccine at least 3 months after the last infusion. Indeed, for mRNA approved SARS-CoV-2 vaccines a 3-week interval between the first and second dose is necessary, then further 4–6 weeks are needed to develop an adequate immunization before ocrelizumab re-administration.

To date, no relevant studies on maintenance and/or generation of immune responses have been reported in MS patients on oral cladribine. Considering the complex mechanism of action of cladribine, investigating immune response to vaccines at different timepoints after treatment could be useful. Following cladribine administration, a rapid depletion of both T and B peripherally circulating lymphocytes is observed, with a mean nadir at 13–24 weeks [46]. Several studies are currently ongoing to test humoral and cell-mediated immune responses during vaccination in patients treated with cladribine. Among these, CLOCK-MS, a 24-month, open-label, randomized, multicenter phase-IV research study identified antibody titers after administration of influenza vaccine in patients treated with cladribine. In particular, increased antibody titers after 4 weeks from vaccination were demonstrated [66]. Furthermore, in MS-Magnify study, aimed at determining the onset of cladribine effect in highly active RRMS patients, it was observed that seroprotection or increased seasonal influenza titers occurred in treated patients who were vaccinated early (month 1.5 to 6 of year 1, and month 1 to 4.5 of year 2 of treatment) or late (month 8.5 to 10.5 of year 1), and that seroprotection was maintained irrespective of total lymphocyte count [67]. Finally, the CLARITY clinical trial, conducted in patients treated with oral cladribine (3.5 mg/kg) vs placebo, has not documented adverse events associated with the administration of vaccines [68]. So it could be reasonable to wait at least 4 weeks after the last course of therapy or, in case of lymphopenia, to wait until the recovery of lymphocyte count.

In summary, currently available data show that vaccinations do not exacerbate MS, provoke a relapse, or prevent DMTs from being effective. Inactivated vaccines are considered safe in patients treated with any disease-modifying drugs, although in some cases may be less effective. In any case, it is not recommended to discontinue or modify DMTs to improve vaccine efficacy, as the risk of disease reactivation and progression outweighs the potential benefit [9]. At the same time, a reduced response is likely to be better than none. Knowledge of vaccine responses on DMTs and a careful risk/benefit assessment is mandatory. If at all possible, vaccinations clearly should be recommended and administered to people with MS.
